# Modular organization and reticulate evolution of the ORF1 of Jockey superfamily transposable elements

**DOI:** 10.1186/1759-8753-5-19

**Published:** 2014-07-01

**Authors:** Cushla J Metcalfe, Didier Casane

**Affiliations:** 1 Universidade de São Paulo, Instituto de Biociências, Rua do Matão 277, Cidade Universitária, São Paulo 05508-090 SP, Brazil; 2 Laboratoire Evolution, Génomes et Spéciation, UPR9034 CNRS, 1 avenue de la terrasse, 91198 Gif-sur-Yvette, France; 3 Université Paris Diderot, Sorbonne Paris Cité, 5 rue Thomas-Mann, 75205 Paris, France

**Keywords:** Long interspersed nucleotide elements, Non-long terminal retrotransposon, Open reading frame 1, Plant homeodomain, RNA recognition motif

## Abstract

**Background:**

Long interspersed nuclear elements (LINES) are the most common transposable element (TE) in almost all metazoan genomes examined. In most LINE superfamilies there are two open reading frames (ORFs), and both are required for transposition. The ORF2 is well characterized, while the structure and function of the ORF1 is less well understood. ORF1s have been classified into five types based on structural organization and the domains identified. Here we perform a large scale analysis of ORF1 domains of 448 elements from the Jockey superfamily using multiple alignments and Hidden Markov Model (HMM)-HMM comparisons.

**Results:**

Three major lineages, Chicken repeat 1 (CR1), LINE2 (L2) and Jockey, were identified. All Jockey lineage elements have the same type of ORF1. In contrast, in the L2 and CR1 lineage elements, all five ORF1 types are found, with no one type of ORF1 predominating. A plant homeodomain (PHD) is much more prevalent than previously suspected. ORF1 type variations involving the PHD domain were found in many subgroups of the L2 and CR1 lineages. A Jockey lineage-like ORF1 with a PHD domain was found in both lineages. A phylogenetic analysis of this ORF1 suggests that it has been horizontally transferred. Likewise, an esterase containing ORF1 type was only found in two exclusively vertebrate L2 and CR1 groups, indicating that it may have been acquired in a vertebrate common ancestor and then transferred between the lineages.

**Conclusions:**

The ORF1 of the CR1 and L2 lineages is very structurally diverse. The presence of a PHD domain in many ORF1s of the L2 and CR1 lineages is suggestive of domain shuffling. There is also evidence of possible horizontal transfer of entire ORF1s between lineages. In conclusion, while the structure of the ORF2 appears to be highly constrained and its evolution tree-like, the structure of the ORF1 within the CR1 and L2 lineages is much more variable and its evolution reticulate.

## Background

Transposable elements (TEs) are mobile genetic elements found in nearly all eukaryotic genomes and are the major contributor to variation in genome size [1]. They are genomic ‘invaders’, one type of genomic component involved in genomic conflict with the host genome. There is an increasing body of evidence suggesting that the evolution of TEs is reticulate [2-6]. For example, the envelope domain has been independently acquired by three Gypsy lineages [4].

**Figure 1:**
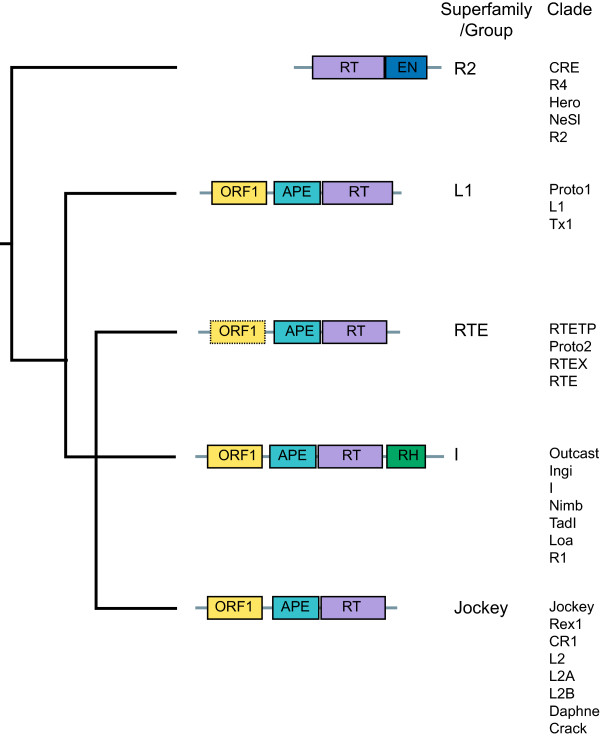
**LINE superfamilies.** Relationships between LINE superfamilies/groups and assignment of clades to superfamilies/groups based on reverse transcriptase (RT) phylogeny [7,9,10]. LINE clades were first assigned to five groups (R2, L1, RTE, I and Jockey) by Eickbush and Malik [8]. Groups are called superfamilies in the TE classification paper by Wicker *et al*. [7]. The ORF1 is not present in some RTE clades (shown with a dashed outline). In this paper, all Jockey superfamily/group full-length sequences from the Repbase database were assigned to three lineages based on an APE-RT phylogeny (see Figure 3). Subgroups were identified within the three lineages and ORF1 structures (see Figure 2) mapped onto the phylogeny (see Figures 4, 5 and 6).

LINEs are the predominant order of elements found in most animal genomes examined [7]. Fourteen clades were assigned to five groups based on reverse transcriptase (RT) phylogeny by Eickbush and Malik [8]. These five groups were converted to superfamilies in the TE classification system proposed by Wicker *et al*. [7]. Since then, several clades and one group has been added [9]. Here we use the term superfamily/group since neither term is universally accepted. The Jockey superfamily/group is one of the younger superfamilies/groups that encodes for an apurinic endonuclease (APE) within the ORF2 [8]. Eight clades fall within the Jockey superfamily/group, Jockey, Rex1, CR1, L2, L2A, L2B, Daphne and Crack (Figure 1) [9]. The CR1 and L2 clades are widely distributed in metazoans, while the Jockey clade is confined to the arthropods.

**Figure 2:**
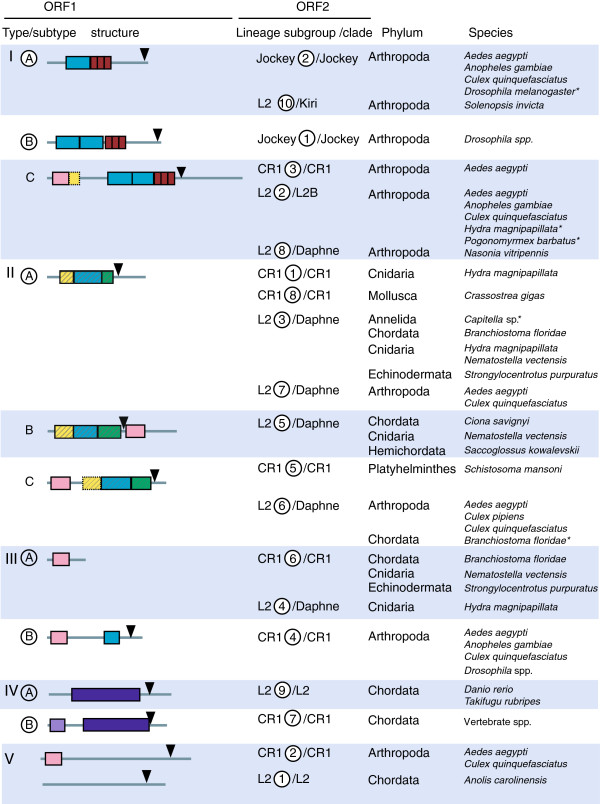
**ORF1 types identified in the Jockey, CR1 and L2 lineages.** Subtypes (A, B, C) are used to show the diversity of ORF1 structures within types identified in this paper. Subtype titles within a circle denote those previously described by Khazina and Weichenrieder [11] and Kapitonov *et al*. [16]. Lineages and subgroups were identified by ORF1 structure and phylogenetic structuring based on the apurinic endonuclease (APE) and reverse transcriptase (RT) domains (see Figures 3, 4). Clades within lineages were identified by the RTclass1 tool [9]. The phylum and species are taken from the Repbase sequence title [17]. The ORF1 structure schematic shows coding domains 5’ to the endonuclease identified in this publication and are drawn to scale. Domains not always present are shown with a dashed outline. Red: CCHC, gag-like Cys_2_HisCys zinc-knuckle; green: CTD, C terminal domain; yellow: coiled-coil domain; purple: esterase; pink: PHD, plant homeodomain; blue: RRM, RNA recognition motif; lilac: zf/lz, zinc finger/leucine zipper. The hatched CC, RRM + CTD domains indicate transposase 22, the RCSB Protein Data Bank entry 2yko and Pfam entry PF02994. A key to all the domains is shown in Figure 6.

All known LINE ORF2s code for a RT and an endonuclease. The APE and RT proteins supply the enzymatic activities for cDNA synthesis and host genome nicking during the replication cycle. The structure and function of the ORF1 is less well understood and the structure more variable. Khazina and Weichenrieder [11] have classified ORF1s into five types based on the type and organization of domains (Figure 2). Type I contains at least one RNA recognition motif (RRM) immediately upstream of a Cys_2_HisCys (CCHC) zinc knuckle, and is found in the I, Jockey, R1, Tad1, plant LINE1 (L1) and L2 clades [11-13]. Type II is the L1 type of ORF1, a coiled-coil (CC) domain [14] upstream of a single RRM domain and a C-terminal domain (CTD) [11,15]. This type is found in the L1, CR1, L2 and R1 clade elements [11,12]. Type III [11] has an occasional C-terminal RRM in addition to the PHD domain. The PHD domain was first identified in CR1 elements [16]. The Type III ORF1 with an RRM and a PHD domain has been found only the CR1 clade. Type IV has an esterase domain and was also first described in CR1 clade elements [11,16] but has also been identified in L2 clade elements [12]. Type V is unclassified ORF1s [11].

The ORF1 classification is based on a sample of 14 ORF1s from 10 LINE clades [11]. Clade allocation was based on the Repbase sequence title, which theoretically indicates the clade that the element belongs to [17]. Here we explore the structure and evolution of the ORF1s of Jockey superfamily/group elements in more depth within a phylogenetic framework. We used all full-length Jockey superfamily/group sequences from the Repbase database [18] for two reasons. First, Repbase is the most comprehensive and widely used TE database. Second, many entries are consensus sequences, allowing us to examine a wide range of elements. We examined 448 full-length Jockey superfamily/group elements. ORF1 structures were determined by multiple alignment and HMM-HMM comparison against three protein databases. The structures were then mapped onto an APE and RT phylogeny. We identified ORF1 types in clades where they had been not previously described. We also identified structural variations of the ORF1 types. We propose that there has been ORF1 domain shuffling in Jockey superfamily/group elements, and that in some instances entire ORF1s may have been horizontally acquired.

## Results

### Sequence retrieval from Repbase and Repeatmasker classification

One thousand two hundred forty nine Jockey superfamily/group sequences from the Jockey, Rex1, CR1, L2, L2A, L2B, Daphne, and Crack clades were downloaded from the Repbase database [18]. These were classified by Repeatmasker as 536 CR1, 422 L2, 54 RexBarber, 206 Jockey, 20 L1 and 1 R1 type sequences. The L1 and R1 sequences were removed. Only one complete RexBarber sequence was found, so this was also removed. After aligning and removing all incomplete sequences, 451 sequences remained: 235 CR1, 87 Jockey and 129 L2 type sequences. Three sequences that did not fall clearly into a subgroup (see next section) - one sequence in the CR1 lineage and two in the L2 lineage - were not further analyzed.

### Phylogenetic analysis, clade assignment and ORF1 domains identified

**Figure 3:**
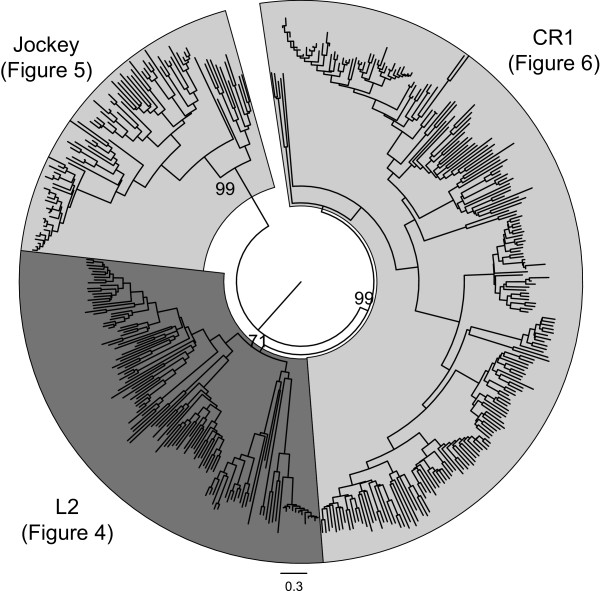
**Full-length Jockey superfamily elements fall into three lineages: CR1, L2 and Jockey.** The neighbor-joining phylogeny is based on a concatenation of the ORF2 apurinic endonuclease (APE) and reverse transcriptase (RT) domains and inferred using MEGA 6 [20] with the Jones-Taylor-Thornton (JTT) substitution matrix. Robustness of the nodes was estimated by 500 bootstrap replications. Only bootstrap values for the three lineages are shown. Lineages are delineated by alternate light and dark grey shading using FigTree [21]. Details of the ORF1 types identified in the three lineages are shown in Figures 4, 5 and 6.

**Figure 4:**
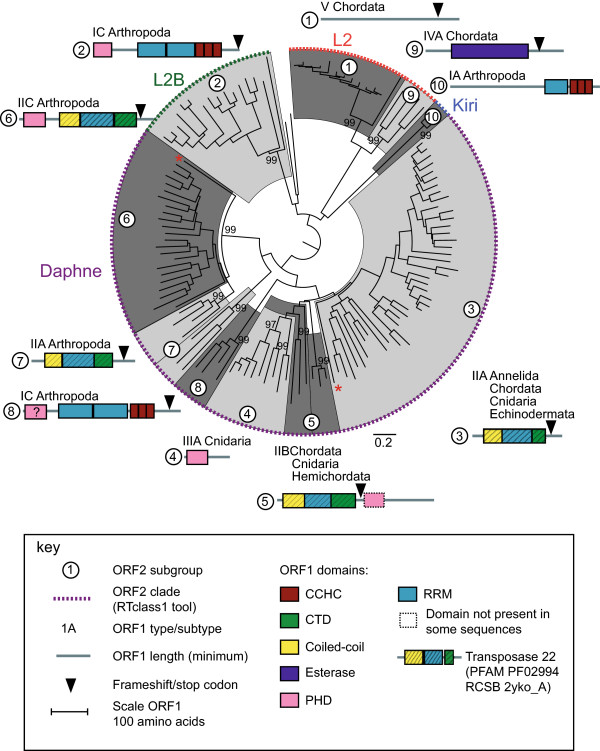
**ORF1 types mapped onto the L2 lineage apurinic endonuclease (APE)-reverse transcriptase (RT) phylogeny.** Please see the legend for Figure 6 for more details.

**Figure 5:**
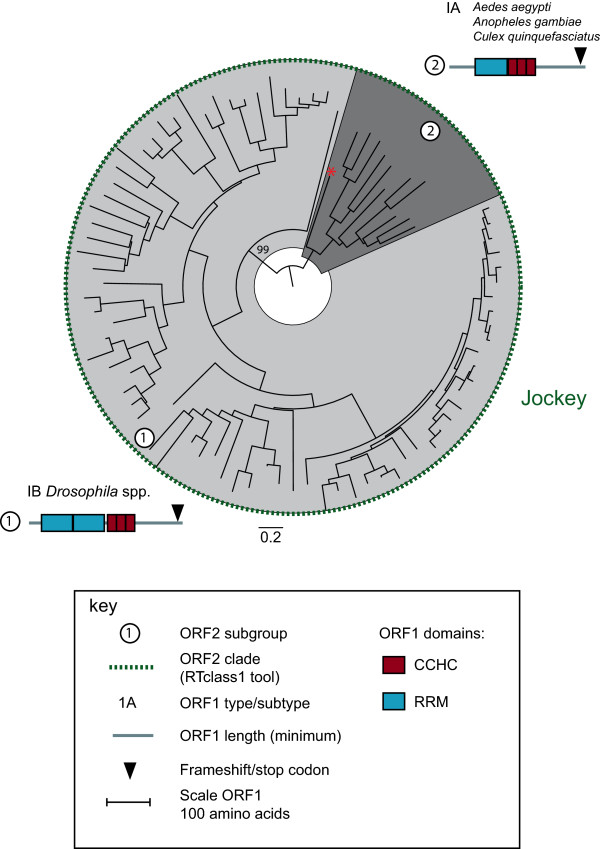
**ORF1 types mapped onto the Jockey lineage apurinic endonuclease (APE)-reverse transcriptase (RT) phylogeny.**

**Figure 6:**
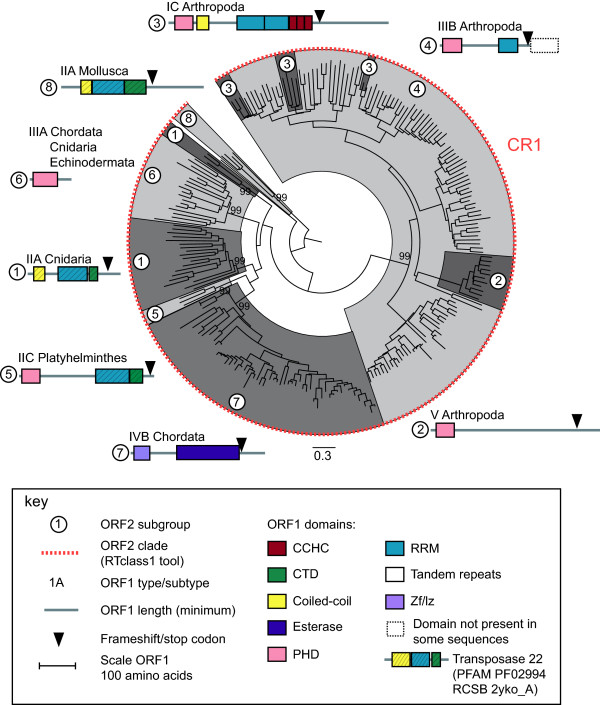
**ORF1 types mapped onto the CR1 lineage apurinic endonuclease (APE)-reverse transcriptase (RT) phylogeny.** The phylogenies are sub-trees of the Jockey superfamily APE-RT (ORF2) phylogeny (see Figure 3). Subgroups were identified based on phylogenetic clustering and the ORF1 type. These are delineated by alternate light and dark grey shading using FigTree [21] and numbered. Subgroup numbers are shown within white circle in the phylogeny and next to the ORF1 structure schematic. Subgroups were assigned to clades using the RTclass1 tool [9]. The ORF1 type/subtype (see Figure 2) and phylum in which elements were identified are shown above the schematic. The phylum is not shown if only a single sequence was identified. ORF1 domains were identified by multiple alignment of all sequences within the subgroup and an HMM-HMM comparision [22]. Coiled-coil domains were identified using Pcoils [23]. The ORF1 structure schematic shows domains 5’ to the endonuclease. The ORF1 structure is drawn to scale, domain lengths are the minimum identified. We have used the term ‘ORF1’ for simplicity’s sake, although in some cases domains are shown that are probably at the 5’ end of the ORF2 (L2 subgroup 5 and CR1 subgroup 4) or at the 5’ end of a single ORF (L2 subgroup 4 and CR1 subgroup 6). Domains are color-coded, details are shown in the key. CCHC, gag-like Cys_2_HisCys zinc-knuckle; CTD, C terminal domain; PHD, plant homeodomain; RRM, RNA recognition motif; zf/lz, zinc finger/leucine zipper. Transposase 22 refers to the RCSB Protein Data Bank entry 2yko_A and Pfam entry PF02994, the L1ORF1 protein composed of a coiled-coil, RRM and CTD domain [24]. Red asterisks indicate single sequences within a subgroup from a different phylum. In Figure 4 (L2 lineage) these are a single *Branchiostoma floridae* (Chordata) sequence in subgroup 6 and a single *Capitella* species (Annelida) sequence in subgroup 3. In Figure 5 (Jockey lineage) this is a single *Drosophila* sequence in subgroup 2.

The sequences fell into three well-supported lineages - L2, CR1 and Jockey - except perhaps for the L2 lineage, which has a bootstrap value of 71. These lineages are consistent with the ‘type’ classification by Repeatmasker based on TE encoded proteins (Figure 3) [19]. Within each lineage, subgroups were identified both by the level of bootstrap support in the phylogenetic analysis and by the type of ORF1 domains found (Figures 4, 5 and 6). Subgroups were named according to the lineage identified and a subgroup number assigned. Several subgroups are not monophyletic but have been grouped together based on ORF1 structure. The Wicker *et al*. [7] classification system proposes that sequences belong to the same family if they share 80% nucleotide sequence identity in at least 80% of the coding or internal domain, or within their terminal repeats, or in both. Apart from the L2 subgroup 1, the subgroups we identified share 49 to 70% identity in the RT domain and so are not equivalent to a family (Table 1). We have used the term ‘lineage’ to describe the three large grouping identified by phylogenetic analysis, and the term ‘subgroup’ to describe the groups within lineages identified by phylogenetic analysis and ORF1 structure. L2 subgroup 2 therefore refers to the L2 lineage subgroup 2, not the L2 clade. When referring to the L2 clade, we will use the term ‘clade’. This was to avoid confusion with the Repbase clades. We chose not to use the term ‘type’ used by Repeatmasker to avoid confusion with ORF1 types.

**Table 1:** Identification of ORF1 domains

			**ORF1**						
**Lineage/subgroup**^**a**^	**No. Seqs**	**Av. RT nt% pairwise identity**^**b**^	**Type/subtype**^**c**^	**Domain**^**d**^	**Length aa**^**e**^	**Av. aa% pairwise identity**^**f**^	**Top hit**^**g**^	**Prob**^**h**^	**No. RRMs/CCHCs**
L2_1	13	82.2	V	No hits					
L2_2	16	58.2	IC	PHD	50	30.0	3zpv_A	98.2	
				RRM	155	26.0	2ghp_A	80.7	2
				CCHC	67	46.1	PTHR23002	98.5	3
L2_3	43	57.6	IIA	Tnp22	158	30.6	2yko_A	100.0	1
L2_4	7	64.8	IIIA	PHD	51	43.0	2vpb_A	99.7	
L2_5	8	52.2	IIB	Tnp22	208	27.7	2yko_A	100.0	1
				PHD	55	29.1	3lqh_A	99.7	
L2_6	23	55.9	IIC	PHD	51	42.5	2vpb_A	96.6	
				Tnp22	209	35.5	2yko_A	100.0	1
L2_7	7	50.3	IIA	Tnp22	191	21.7	2yko_A	100.0	1
L2_8	4	62.7	IC	RRM	188	34.5	3smz_A	86.7	2
				CCHC	60	45.3	PTHR23002	99.2	3
L2_9	4	57.1	IVA	Esterase	176	28.0	3p94_A	99.9	
L2_10	2	79.0	IA	RRM	63	85.9	2lkz_A	90.2	
				CCHC	64	76.9	PTHR23002	98.9	
Jockey_1	75	51.8	IB	RRM	143	29.1	2cjk_A	96.6	2
				CCHC	55	46.2	PTHR23002	98.9	3
Jockey_2	12	51.6	IA	RRM	74	32.5	2lxi	93.8	1
				CCHC	54	39.4	PTHR23002	98.8	3
CR1_1	22	53.5	IIA	Tnp22	186	23.5	2yko_A	98.5	1
CR1_2	11	70.0	V	PHD	50	41.3	2vpb_A	94.6	
CR1_3	8	58.8	IC	PHD	53	48.1	2vpb_A	96.3	
				CC	34	22.3			
				RRM	144	37.1	1b7f_A	93.8	2
				CCHC	65	46.8	PTHR23002	98.3	3
CR1_4	112	53.1	IIIB	PHD	53	27.6	3zpv_A	95.0	
				RRM	53	28.4	2dhg_A	79.4	1
CR1_5	3	58.1	IIC	PHD	50	71.9	2vpb_A	99.4	
				Tnp22	129	41.8	2yko_A	63.1	1
CR1_6	18	54.0	IIIA	PHD	48	40.6	1wep_A	99.4	
CR1_7	56	62.9	IVB	lz	44	34.0	2yon_A	85.1	
				zf	44	34.0	2gmg_A	37.1	
				Esterase	174	43.5	2waa_A	99.7	
CR1_8	4	61.5		Tnp22	175	36.5	2yko_A	100.0	

^a^Lineage and subgroup identified by phylogenetic analysis based on a concatenation of the ORF2 apurinic endonuclease (APE) and reverse transcriptase (RT) domains. For further details please see the text.

^b^Average percent pairwise nucleotide identity of the RT domain for each subgroup, estimated using Geneious [25].

^c^ORF1 type (I-V) identified for each subgroup, based on ORF1 types described by Khazina and Weichenrieder [11]. Subtypes (A, B and C) are used to show the diversity of ORF1 structures within types identified in this paper.

^d^Domains identified within the ORF1 by HMM-HMM comparision [26] or by Pcoils [23]. CC, coiled-coil domain; CCHC, gag-like Cys_2_HisCys zinc-knuckle; lz, leucine zipper; PHD, plant homeodomain; RRM, RNA recognition motif; Tnp22, transposase 22, (RCSB Protein Data Bank entry 2yko_A and Pfam entry PF02994), which is the L1ORF1 protein composed of a coiled-coil, RRM and CTD domain [24]; zf, zinc finger.

^e^Minimum length of the inferred amino acid sequence for each domain.

^f^Average percent pairwise inferred amino acid identity for each domain, estimated using Geneious [25].

^g^Top hits starting with ‘PTHR’ are from the Panther Classification System, all other top hits are from the RCSB Protein Data Bank.

^h^Probability reported by HHPred [26].

The lineages and subgroups identified were compared with the clade assignment based on the Repbase sequence name and the RTclass1 tool [9]. Clade assignments were concordant with our phylogenetic analysis and Repeatmasker type except for the L2 sequences (Figure 3). These were split into four clades, Daphne, Kiri, L2 and L2B by the RTclass1 tool. The Repbase sequence names did not always reflect clade assignments [see Additional file 1].

### ORF1 classification

The beginning of the ORF1 was identified in all sequence alignments except for two of the four L2 subgroup 8 sequences, which are also lacking the 5’ untranslated region (UTR). Three main domains were identified, a gag-like CCHC domain, an RRM motif and a PHD (Table 1). A sequence logo of the PHD and CCHC domains for all sequences in which they were found is shown in Additional file 2. Alignments for two examples of RRM domains (CR1 subgroup 3 and L2 subgroup 6) are shown in Additional file 3. The number of sequences in each subgroup from the three lineages, that is, CR1, L2 and Jockey, and the domains identified, are summarized in Table 1. Pairwise identity at the amino acid level for the ORF1 domain sequence alignments range from 21.7 to 85.9% and probabilities from 37.1 to 100% (Table 1). Only four domains have probabilities less than 85%, the RRM domain in the L2 subgroup 2, the zinc finger in the CR1 subgroup 7, the RRM domain in CR1 subgroup 4 and the RRM + CTD domain in CR1 subgroup 5.

Five ORF1 types were identified by Khazina and Weichenrieder [11] using a sample of 14 ORF1s from 10 LINE clades. Using as a basis the ORF1 structure described and the elements used by Khazina and Weichenrieder [11], we classified the ORF1s identified in this study into the same types, but have also added a subtype category A, B and C to describe variations (Figure 2). We have classified the ORF1 from the CR1 subgroup 2 as type V, which is an unclassified ORF1, because we were able to identify less than 10% of the entire ORF. Type I has at least one RRM domain immediately upstream of a CCHC zinc knuckle [11]. In our analysis, this ORF1 type was found in all Jockey lineage elements, CR1 subgroup 3 and L2 subgroups 2, 8 and 10 (Figures 2, 4, 5, 6 and Table 1). Type II is found in the human L1 element and has a CC domain, a single RRM domain and a CTD domain [11]. In the Research Collaboratory for Structural Bioinformatics (RCSB) and the Protein families (Pfam) databases the three domains are submitted as a single entry, transposase 22 (2yko_A and PF02994, respectively). This type was identified in several CR1 and L2 subgroups (Figures 2, 4, 6 and Table 1). In the L2 subgroup 5 a PHD domain was found downstream from the transposase 22, after a stop and start codon and therefore is probably at the beginning of the ORF2. A PHD domain was also identified in L2 subgroup 6 and CR1 subgroup 5, at the N-terminus of the ORF1 (Figures 2, 4, 6 and Table 1). For Type III, Khazina and Weichenrieder [11] predicted an occasional C-terminal RRM in addition to the PHD domain. A single PHD domain was found by us in CR1 subgroup 6 and L2 subgroup 4 and an RRM domain associated with an PHD domain in CR1 subgroup 4. Type IV is an ORF1 with an esterase domain [16], sometimes associated with a zinc finger/leucine zipper [16], and was identified in CR1 subgroup 7 and L2 subgroup 9.

### ORF1 Clans clustering and phylogenetic analysis of Type I ORF1 domains

**Figure 7:**
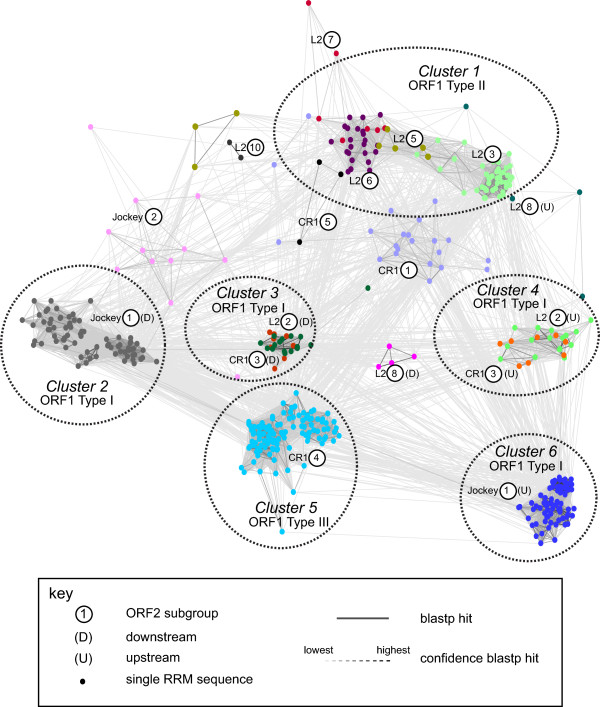
**RNA recognition motif (RRM) domains fall into six clusters.** All RRM domains were clustered using CLANS with Blastp and default values [28]. Where two RRM domains were identified, the 5’ domain is labeled ‘U’ for upstream, the 3’ domain ‘D’ for downstream. Single dots are single sequences and are color-coded by subgroup. ORF2 subgroup numbers are shown in circles. Dotted lines connecting sequences represent the confidence in the Blastp hit and are colored from dark to light grey; lightest is the lowest, darkest is the highest.

**Figure 8:**
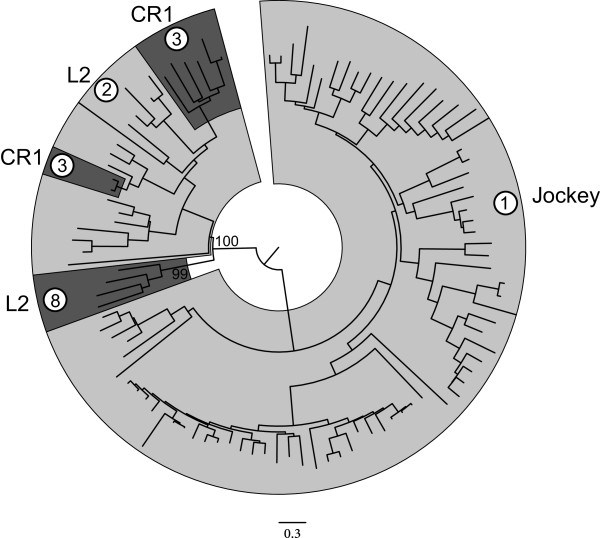
**Neighbor-joining phylogeny based on ORF1 Type I domains.** The ORF1 of CR1 group 3 sequences cluster with those of L2 group 2, suggesting that this type of ORF1 may have been horizontally acquired across lineages. The phylogeny was estimated using MEGA 6 [20] and inferred using the JTT substitution matrix. The robustness of the nodes was estimated by 1,000 bootstrap replicates. Only bootstrap values for major groups are shown.

The PHD and CCHC domains are quite small, 50 or so amino acids long [see Additional file 2]. An attempt was made to determine the relationship between PHD and CCHC domains from different subgroups by multiple alignments using muscle [27] and a phylogenetic analysis using MEGA 6 [20]. This resulted in trees for both the PHD and the CCHC domains with unresolved branches (data not shown). A Clans analysis [28], which is multiple alignment independent, using all PHD and CCHC domains, resulted in two large clusters, one for the CCHC domains and one for the PHD domains. No clear structuring was found within these two clusters.Six clusters were inferred in a Clans clustering of the individual RRM domains (Figure 7). Cluster 1 consisted of ORF1 type II RRM domains from CR1 subgroup 5 and L2 subgroups 3, 5 and 6. All other ORF1 type II RRM domain sequences did not cluster strongly with other subgroups. The ORF1 type III RRM domain was found in a single subgroup, CR1 subgroup 4. These sequences all fell into a single cluster, Cluster 5. The ORF1 type I RRM domains fell into 4 clusters. The upstream (‘U’) and downstream (‘D’) RRM domains from the Jockey subgroup 1 formed two separate clusters (Clusters 2 and 6). The RRM domains from L2 subgroup 2 and CR1 subgroup 3 fell into two clusters, the upstream RRM domains together in a single cluster (Cluster 4), and the downstream RRM domains together in a separate cluster (Cluster 3).The relationship between the type I ORF1 domains is shown in Figure 8. The ORF1 domains from the Jockey subgroup 1 and from L2 subgroup 8 fell into two well-supported groups. The ORF1 domains from CR1 subgroup 3 and L2 subgroup 2, however, clustered together, with the CR1 subgroup 3 sequences being embedded at two positions within the L2 subgroup 2-sequence phylogeny.

## Discussion

In most LINE superfamily/group elements there are two ORFs, in which the ORF2 codes for at least two domains, an APE and RT **(**Figure 1). In contrast to the ORF2, the structure of the ORF1 is not only less well characterized but also more structurally variable. Based on a sample of 14 ORF1s from 10 LINE clades, ORF1s have been previously classified into five types, depending on the organization and type of domain present [11] (Figure 2). Elements from the Jockey superfamily/group exhibit the highest ORF1 diversity. This diversity is chiefly found in the CR1 elements, in which three of the five types have been identified [11]. A large scale analysis of the ORF1 of Jockey superfamily/group elements has not been previously attempted. Here we map the structure of the ORF1 from 448 Jockey superfamily/group elements onto a phylogenetic framework.

### ORF2 phylogenetic and clade analysis

Full-length elements from the eight clades of the Jockey superfamily/group, Jockey, Rex1, CR1, L2, L2A, L2B, Daphne and Crack, were assigned by phylogenetic analysis to three well supported lineages, L2, CR1 and Jockey (Figure 3). This assignment is consistent with the ‘type’ classification by Repeatmasker [19] [see Additional file 1]. Elements were further assigned to clades using the RTclass1 tool [9]. Repbase sequence names theoretically reflect the clade that they are assigned to [17]. Clade assignments were concordant with our phylogenetic analysis and Repeatmasker type except for the L2 sequences (Figure 3). These were split into four clades, Daphne, Kiri, L2 and L2B, by the RTclass1 tool (Figure 4). For these four clades, the Repbase sequence names did not consistently reflect clade assignments [see Additional file 1].

### Diversity in ORF1 domains and structure

The ORF1s of the L2 and CR1 elements were found to be highly diverse, both in terms of structure and the number of types of ORF1s found (Figures 2, 4 and 6). All five ORF1 types [11] were identified in the L2 and CR1 lineages, in contrast, all elements in the Jockey lineage have a single type of ORF1 (Figure 5). Three structural variations of ORF1 types I and II [11] were identified that contained a PHD domain (Figure 2). A total of eight differently structured ORF1s were found in the L2 lineage, and seven in the CR1 lineage. While the type I and II ORF1s predominate in the L2 lineage and the ORF1 type III B was only found in the CR1 lineage, there is no clear ‘CR1-like’ or ‘L2-like’ ORF1.

For the ORF1 types II and III, the type classification is somewhat concordant with a clustering analysis of the RRM domains (Figure 7) and the top hits from the HMM-HMM analysis (Table 1). However the RRM domains from type I do not all cluster together and the top hits are not the same, suggesting similarity at the structural but not amino acid sequence level (Figure 7). A major homology region (MHR) has been previously identified in the TART, TAHRE and DOC elements of the Jockey lineage [29]. In our analysis, these elements have a type IB ORF1 [see Additional file 1]. A visual comparison of the amino acid alignment of the MHR in the TART and DOC elements of the Jockey lineage [30] with our alignment of the RRM domain identifies the MHR as a RRM domain (data not shown).

### Functions and putative functions of ORF1 domains

Current evidence suggests that the RRM, esterase and CCHC zinc-knuckle domains are all involved in transcript binding, stabilization and chaperoning. The L1 ORF1 RRM domain is a single-stranded nucleotide binding protein with nucleic acid chaperone activity, preferentially binding to RNA [24,31]. Many RNA binding proteins have a modular structure, and the RRM domain itself is often found in multiple copies [32], as in the type I ORF1s (Figure 2). The CTD domain has been shown experimentally to assist the RRM domain in nucleic acid binding [24]. The CTD and CCHC domains therefore probably act as accessory domains in RNA binding. In the type I ORF1s, the CCHC zinc-knuckles found are gag-like, a gene found in long terminal repeat-retrotransposons and retroviruses. In the HIV retrovirus the role of the CCHC domain has been demonstrated to include the chaperoning of the transcript as well as the full-length cDNA [33]. Consistent with these findings, in SART1, a telomeric specific LINE R1 element, all three CCHC zinc-knuckle motifs are involved in the specific packaging of the mRNA into the ribonucleoprotein (RNP) complex [34]. TART elements are Jockey clade elements that form the telomeres in *Drosophila* and therefore presumably perform an essential host cellular function. Intriguingly, RNP complexes for TART were found to be efficiently transported into the nucleus, unlike non-telomeric Jockey clade elements [30], suggesting that there may be a host control system of ‘friendly’ and ‘unfriendly’ RNP complexes. The structure of the esterase domain has been recently elucidated [35]. The authors suggest that the esterase domain is involved in membrane targeting, maybe driving RNP assembly on membrane surfaces [35]. As far as we can tell, there have been no functional studies specifically on the PHD domain in LINE elements. However the PHD domain in other proteins has been well-studied and have been shown to recognize modified histones [36,37]. Domains in this class are known as ‘epigenetic readers’. Although there are examples of LINEs that target specific genome regions, such as tRNA genes, telomeres or microsatellites, in most LINEs with an APE domain the target specificity of host sequences has been relaxed [10,38,39]. This suggests that the PHD domain may be involved in general targeting of the host genome during integration. In some subgroups there is apparently a single ORF, with a PHD domain at the N-terminus (L2 subgroup 4 and CR1 subgroup 6). The 5’ UTR of LINE elements is widely variable [38], so it is difficult to generalize about their structure. However, some of these elements are reported by Repbase as ‘autonomous’ and the region 5’ to the PHD domain is highly repetitive, suggesting that these may be full-length elements. These elements may therefore be a reversion to R2 like elements, with a single ORF or may be TE parasites, using machinery of other elements to transpose.

### Reticulate evolution and horizontal ORF1 acquisition

The possibility of horizontal ORF1 acquisition has been proposed to explain, for example, the presence of the esterase type ORF1 in elements from diverse phyla in phylogenetically disjunct LINE clades [35]. In our analysis the esterase type ORF1 was found only in two exclusively vertebrate subgroups, the L2 subgroup 9 and CR1 subgroup 7. This suggests that in the Jockey superfamily/group this ORF1 type may have been acquired in a vertebrate common ancestor and then transferred between the lineages. Our results also suggest that the ORF1 of CR1 subgroup 3 and L2 subgroup 2 may also have been horizontally transferred, possibly within a mosquito host. This ORF1 has three CCHC zinc fingers downstream from two RRM domains. In a clustering analysis of individual RRM domains, the upstream RRM domains from CR1 subgroup 3 and L2 subgroup 2 cluster together (Cluster 4 in Figure 7), while the downstream RRM domains cluster together in a separate subgroup (Cluster 3 in Figure 7). In a phylogenetic analysis of all five domains, the two RRM and three CCHC domains, from all sequences with this type of ORF1, CR1 subgroup 3 sequences cluster with those of L2 subgroup 2 (Figure 8). All CR1 subgroup 3 sequences are from the mosquito, *Aedes aegypti*, and all except one sequence in L2 subgroup 2 are from mosquitoes, including *Aedes aegypti* (Table 1). All other CR1 mosquito sequences (subgroup 4) have the type III ORF1. We therefore speculate that the ORF1 has been horizontally acquired within a mosquito host. Recombination at the DNA or RNA level is one way in which ORF1s may be horizontally acquired [35]. Due to their mode of replication, LINEs are often 5’ truncated upon insertion. This suggests a simple way an ORF1 may become associated with an un-related ORF2, resulting in ORF1 shuffling. If a TE is 5’ truncated in such a way that it has a complete ORF2 but no ORF1 and the insertion occurs into other type of TE in between the ORF1 and ORF2, this would result in a hybrid TE with the ORF1 of one type of TE and the ORF2 of another type of TE.

### Modular organization and domain shuffling

A protein domain can be defined as an independent evolutionary unit that can either have an independent function or contribute to the function of a multidomain protein. The major molecular mechanism that leads to multidomain proteins and novel combinations is non-homologous recombination, sometimes referred to as ‘domain shuffling’ [40]. The variability in domain type and organization in ORF1s identified here in the Jockey superfamily/group is also suggestive of domain shuffling. Within ORF1 types the chief difference we identified (Figure 2) is the variable presence and position of a PHD domain in the CR1 and L2 lineage elements. From our data, we cannot determine the direction of domain shuffling. The RRM and CCHC domains are found in the ORF1 of L1, I and Jockey superfamily/group elements [11,13] indicating that they are ancient components of LINEs. The variability in ORF1 structure that is the result of the combination of various modules, seen here in Jockey superfamily/group elements, is concordant with an increasing body of data indicating that the origin and evolution of TEs is reticulate, that is, it involves extensive domain shuffling [2-6].

## Conclusions

We inferred a phylogeny based on the APE and RT domains for full-length Jockey superfamily/group elements from the Repbase database. ORF1s structures were mapped onto the ORF2 phylogeny. All Jockey lineage elements have the same type of ORF1, with one to two RRM domains upstream of three CCHC domains. In contrast, in the L2 and CR1 lineage elements, all five ORF1 types are found, with no one type of ORF1 predominating. The structure of these ORF1s is indicative of domain shuffling. The PHD domain is much more prevalent than previously suspected; it was identified in four ORF1 types in many subgroups within the L2 and CR1 lineages and both upstream and downstream of the RRM domain. There was also evidence of reticulate evolution and possibly horizontal transfer of entire ORF1s. The ORF1 of the CR1 subgroup 3 and L2 subgroup 2 is unusual, a Jockey like ORF1 with a PHD domain upstream of the RRM domains. Our analyses suggest that this ORF1 has been horizontally transferred. From our data we could not determine the direction or origin of this transfer. The esterase domain type ORF1 was found only in two exclusively vertebrate subgroups from the L2 and CR1 lineages, indicating that it has been acquired in a vertebrate common ancestor and then may have been transferred between the lineages. Within the Jockey superfamily/group, while the structure of the ORF2 appears to be highly constrained and its evolution tree-like, the ORF1 structure of the L2 and CR1 lineages is much more variable and its evolution reticulate.

## Methods

### Sequence retrieval from Repbase, Repeatmasker classification and alignment

All 1,249 sequences from the Jockey superfamily/group [9] were downloaded from the Repbase database in April 2014 [18]. The two lungfish sequences were taken from Metcalfe *et al*. [41]. The sequences were classified into ‘type’ by screening against a database of transposable element encoded proteins as implemented by the web-based Repeatmasker program [19]. Sequences were then conceptually translated, aligned using ClustalW as implemented in BioEdit and adjusted by eye [42]. Incomplete sequences were removed.

### Phylogenetic analysis and identification of subgroups

The ORF2 RT domain is typically used to classify TEs at both the superfamily/group and clade levels [7,9]. Phylogenies based on the APE are generally concordant with RT phylogeny, but with less resolution [10]. We therefore inferred two phylogenies, one based on the RT domain alone, and one based on a concatenation of the APE and RT domains. For both regions, the optimal model of amino acid substitution was estimated using MEGA 6 [20] with default settings. A neighbor-joining tree was inferred using the highest-ranked substitution model (JTT matrix) and the robustness of the nodes estimated by 500 bootstrap replicates. The topology of the two trees was similar. The chief difference between the two was that in the tree based on APE and RT domains the sequences fell into three well-defined groups consistent with the Repeatmasker ‘type’ classification, whereas in the tree based on the RT domain alone, the Repeatmasker L2 ‘type’ sequences fell into two groups with poor bootstrap support for the relationship between the groups (data not shown). All subsequent analyses were therefore based on the tree inferred from a concatenation of the APE and RT domains.

Subgroups within the three large ‘type’ groups were identified based on ORF1 alignment and support by the phylogenetic analysis. Sequences were renamed according to the type identified by Repeatmasker and subgroup. The RTclass1 tool [9] was used to classify subgroups into clades. Because the RTclass1 tool allows the analysis of a single sequence at a time, at least two sequences from each subgroup were assigned to a clade. Percent pairwise identity within the reverse transcriptase at the amino acid level for both the lineages and the clades were estimated using Geneious [25].

### Open reading frame 1 analysis

For each subgroup identified the region 5’ to the endonuclease domain and 3’ to the 5’ UTR was extracted as an alignment. For simplicity’s sake this region will be referred to ‘ORF1’, although some domains identified are most likely at the beginning of ORF2 or are at the 5’ end of a single ORF. The beginning of the ORF1 was identified by a methionine and checked against the Repbase EMBL file if the translation was available. Each subgroup was analyzed for similarity to known domains using HMM-HMM comparisons as implemented in HHpred [22] against the following databases, the RCSB Protein Data Bank [43] as at 27 December 2012, the Pfam database [44] as at 2 December 2011 and the Panther Classification System [45] as at 1 May 2012. For each region the top hit was taken as the hit with the highest probability, or the hit with the highest coverage with a high probability (>85%).

For sequences with top hits against the RCSB Protein Data Bank [43], the RCSB record was checked to determine the type of the domain identified. For RRM domains, the publication associated with the top hit at the RCSB Protein Data Bank [43] was used to find the RNP consensus sequences. The JnetPred secondary structure prediction software [46] as implemented in JalView [47] was used to identify beta-sheets and alpha-helices. Pcoils [23] was used to infer coiled-coil domains and to confirm the position of coiled-coil domains in transposase 22 domains. For each subgroup the pairwise percent identity at the amino acid level for the ORF1 was estimated using Geneious [25].

### Clans clustering and phylogenetic analysis of ORF1 domains

Domains identified as RRM were extracted from the ORF1 sequences. The region between the RNP2 and RNP1 consensus sequences was used because this was the only region shared by all sequences. For sequences where two RRM domains were identified, each domain was extracted separately, the first domain labeled ‘U’ for upstream and the second domain labeled ‘D’ for downstream. The RRM domains were clustered using CLANS and Blastp with default values [28].

For subgroups where the ORF1 structure was two RRM domains upstream of three CCHC domains, the entire region containing the RRM and CCHC domains were extracted, aligned using MUSCLE [27] and a neighbor-joining phylogeny inferred using MEGA 6 [20] with the highest ranked substitution model (JTT matrix) .

## Abbreviations

APE: apurinic endonuclease; CC: coiled-coil; CCHC: Cys_2_HisCys zinc-knuckle domains; CR1: chicken repeat 1; CTD: C-terminal domain; HMM: Hidden Markov model; JJT: Jones-Taylor-Thornton; LINE: long interspersed nuclear element; L1: LINE1; L2: LINE2; MHR: major homology region; ORF: open reading frame; PHD: plant homeodomain; Pfam: protein families database; RNP: ribonucleoprotein; RRM: RNA recognition motif; RT: reverse transcriptase; TE: transposable element; UTR: untranslated region; RCSB: Research Collaboratory for Structural Bioinformatics.

## Competing interests

The authors state that they have no competing interests to declare.

## Authors’ contributions

CJM and DC conceived of the study, and participated in its design and coordination and helped to draft the manuscript. CJM performed the analyses. Both authors read and approved the final manuscript.

## Supplementary Material

Additional file 1 **List of all sequences analyzed in this paper, classified according to lineage and subgroup.** Repbase sequences and Australian lungfish sequences from Metcalfe *et al*. [41] used. Lineages and subgroups were identified by our phylogenetic analysis and ORF1 structure analysis. The sequences titles are those used by Repbase [18] or Metcalfe *et al*. [41]. ORF1 types are based on Khazina and Weichenrieder [11]. ORF1 type details are shown in Figures 2, 4, 5 and 6. Repeatmasker type is the type assigned by the web-based Repeatmasker program using a database of transposable element encoded proteins [19]. Clades were assigned using the RTclass1 tool [9]. Click here for file

Additional file 2 **Sequence logos of all PHD and CCHC domains identified.** The domains were aligned using MUSCLE [27]. Sequence logos were created using Weblogo [48]. Click here for file

Additional file 3 **Alignment of the two RRM domains from CR1 lineage subgroup 3 and L2 lineage subgroup 2.** Alpha helices and beta sheets were identified using Jnet secondary structure prediction [49] as implemented by Jalview [47]. Putative RNP1 and RNP2 domains are based on alignment outputs from the HMM-HMM HHpred [26] analysis. Sequence titles are the lineage and subgroup, identified by our phylogenetic analysis and ORF1 structure analysis, followed by the Repbase title. Click here for file
